# Predicting Survival for Veno-Arterial ECMO Using Conditional Inference Trees—A Multicenter Study

**DOI:** 10.3390/jcm12196243

**Published:** 2023-09-28

**Authors:** Julia Braun, Sebastian D. Sahli, Donat R. Spahn, Daniel Röder, Holger Neb, Gösta Lotz, Raed Aser, Markus J. Wilhelm, Alexander Kaserer

**Affiliations:** 1 Departments of Biostatistics and Epidemiology, Epidemiology, Biostatistics and Prevention Institute, University of Zurich, 8001 Zurich, Switzerland; julia.braun@uzh.ch; 2 Institute of Anesthesiology, University and University Hospital Zurich, 8091 Zurich, Switzerland; sebastian.sahli@usz.ch (S.D.S.); donat.spahn@swisspbm.ch (D.R.S.); 3 Department of Anesthesiology, Intensive Care, Emergency and Pain Medicine, University Hospital Würzburg, 97080 Würzburg, Germany; roeder_d@ukw.de; 4 Department of Anesthesiology, Intensive Care Medicine and Pain Therapy, University Hospital Frankfurt, Goethe University, 60596 Frankfurt, Germany; holger.neb@kgu.de (H.N.); goesta.lotz@kgu.de (G.L.); 5 Clinic for Cardiac Surgery, University Heart Center, University and University Hospital Zurich, 8091 Zurich, Switzerland; raed.aser@usz.ch (R.A.); markus.wilhelm@usz.ch (M.J.W.)

**Keywords:** ECLS, VA ECMO, predictors, conditional inference trees, unbiased recursive partitioning, machine learning

## Abstract

Background: Despite increasing use and understanding of the process, veno-arterial extracorporeal membrane oxygenation (VA-ECMO) therapy is still associated with considerable mortality. Personalized and quick survival predictions using machine learning methods can assist in clinical decision making before ECMO insertion. Methods: This is a multicenter study to develop and validate an easy-to-use prognostic model to predict in-hospital mortality of VA-ECMO therapy, using unbiased recursive partitioning with conditional inference trees. We compared two sets with different numbers of variables (small and comprehensive), all of which were available just before ECMO initiation. The area under the curve (AUC), the cross-validated Brier score, and the error rate were applied to assess model performance. Data were collected retrospectively between 2007 and 2019. Results: 837 patients were eligible for this study; 679 patients in the derivation cohort (median (IQR) age 60 (49 to 69) years; 187 (28%) female patients) and a total of 158 patients in two external validation cohorts (median (IQR) age 57 (49 to 65) and 70 (63 to 76) years). For the small data set, the model showed a cross-validated error rate of 35.79% and an AUC of 0.70 (95% confidence interval from 0.66 to 0.74). In the comprehensive data set, the error rate was the same with a value of 35.35%, with an AUC of 0.71 (95% confidence interval from 0.67 to 0.75). The mean Brier scores of the two models were 0.210 (small data set) and 0.211 (comprehensive data set). External validation showed an error rate of 43% and AUC of 0.60 (95% confidence interval from 0.52 to 0.69) using the small tree and an error rate of 35% with an AUC of 0.63 (95% confidence interval from 0.54 to 0.72) using the comprehensive tree. There were large differences between the two validation sets. Conclusions: Conditional inference trees are able to augment prognostic clinical decision making for patients undergoing ECMO treatment. They may provide a degree of accuracy in mortality prediction and prognostic stratification using readily available variables.

## 1. Introduction

Veno-arterial extracorporeal membrane oxygenation (VA-ECMO) represents the ultimate treatment for cardiopulmonary failure [1,2]. Despite its growing adoption and an increased understanding of it, this therapy is still associated with significant morbidity and resource utilization, with notably high mortality rates [3,4]. Precise and personalized survival prediction based on data available prior to insertion has the potential to enhance clinical decision making and ultimately improve patient outcomes.

Numerous studies have explored predictive factors linked to the outcomes of VA-ECMO [5,6,7,8,9,10,11]. However, effective application of these factors to accurately predict survival in clinical practice presents challenges, as it involves data collection and score calculation immediately before the ECMO procedure. This is especially challenging, if not impossible, if the calculation of the score necessitates the use of a computer and the knowledge of how to correctly calculate it—having both the equipment and the expertise to use it is not realistic in a clinical emergency setting. A study by Schrutka et al. revealed that scoring systems for outcome predictions in patients undergoing ECMO after cardiovascular surgery had insufficient discriminatory power, except for the Simplified Acute Physiology Score (SAPS II) and the Survival After Venoarterial ECMO (SAVE) score [12]. Both scores, the SAVE [9] and the SAPS II [13], were developed using multiple regression models, but involve a large number of variables that are not always routinely collected, again rendering them hard to use if time is scarce. So far, only a limited number of publications have addressed the application of advanced statistical principles in order to easily and quickly predict personalized outcomes in critically ill patients receiving ECMO therapy [14,15].

The aim of the present study was to provide an easy-to-use, pencil-and-paper algorithm using the machine learning technique called unbiased recursive partitioning, based on conditional inference trees to develop and validate a prognostic model that can predict the probability of patient survival before the initiation of ECMO therapy. Such a prognostic model should assist clinicians in making decisions prior to ECMO implantation. Since such decisions often have to be made in time-critical situations, an additional goal was to provide an algorithm with concise variables typically available in the resuscitation room. We compared two sets with different numbers of variables, all of which were available just before ECMO initiation.

## 2. Methods

### 2.1. Study Design

The derivation cohort was based on a retrospective ECMO registry of the University Hospital Zurich, Switzerland, a tertiary care referral hospital [16]. Adult patients treated with VA-ECMO between January 2007 and December 2019 with complete follow-up were included. Exclusion criteria were an age of under 18 years and documented refusal of consent. Veno-venous (VV) and hybrid ECLS were also excluded.

The anonymized validation cohorts were retrospectively derived from two independent ECMO centers of the University Hospitals in Frankfurt and Würzburg. The study was reviewed and the requirement for written informed consent was waived by the Cantonal Ethics Commission of Zurich, Switzerland (BASEC No. 2019-01926). This study follows the TRIPOD (Transparent Reporting of a Multivariable Prediction Model for Individual Prognosis or Diagnosis) reporting policy.

### 2.2. Data Collection

Data were collected through a retrospective review of patient records (e.g., medical history, laboratory values, and survival status) and direct export from the clinical information system (e.g., age and sex). The closest laboratory value before VA-ECMO insertion was noted. The maximum tolerated interval of 4 h for blood gas analysis and 24 h for laboratory values was used.

Only variables available prior to VA-ECMO initiation were included and categorized into two sets. First, a simple data set of limited variables immediately available in the emergency situation was defined to see if these variables could provide relatively reliable predictions (small data set, Table 1). Second, a more comprehensive set of variables was assembled (Supplementary Table S1).

We grouped the indications for the VA-ECMO therapy into four categories according to current literature [2,17,18]: postcardiotomy, cardiopulmonary resuscitation, refractory cardiogenic shock, and other. The category “other” included ECMO indications for lung transplantation and expansive thoracic surgery.

### 2.3. Statistical Analysis

#### 2.3.1. Model Development

All analyses were performed in R, version 4.0.5. We used unbiased recursive partitioning based on conditional inference trees to derive the desired decision algorithm. The idea behind this statistical method is based on machine learning, and can be seen as a data-driven approach to find a set of subgroups that are as homogeneous as possible with respect to the clinical outcome of interest.

The method works as follows: Two distinct steps are executed alternately and iteratively until a predefined stopping criterion is reached. In the first step, a test is conducted for each potential predictor out of the set of candidate variables to evaluate its influence on the outcome variable. The resulting *p*-values are corrected for multiple testing. The variable with the strongest association is then selected. If the null hypothesis cannot be rejected for any of the candidate variables, the algorithm is terminated and it is concluded that there is no variable that is sufficiently associated with the outcome.

The second step aims to find the best possible split for the variable selected in step one, i.e., the value that leads to two subgroups that are as distinct as possible with respect to the outcome. This is done by evaluating all possible dichotomous splits to maximize the differences between the two resulting subgroups.

Those two steps are repeated until no additional associated predictor can be found, or until the a priori stopping criterion is reached. Different kinds of criteria can be defined, for example, a minimal number of individuals in each node. If a split would lead to even smaller nodes, the algorithm terminates. This method has been described and discussed extensively [19,20,21] and has been applied in other clinical contexts [22,23].

An alternative to conditional inference trees is the use of so-called conditional inference forests [19,24,25,26], which we calculated to evaluate a potential loss of information in the conditional inference trees. When using this method, an ensemble of several classification trees is calculated based on many random draws from the data set. Predictions are obtained using the mean or majority prediction of the single trees. This usually leads to considerably higher prediction accuracy, but cannot be done by hand using an easily understandable pencil-and-paper system.

Thus, in total, four different classifications are developed and compared: One using the small data set with data that are immediately available before ECMO implantation, and one using the more comprehensive data set described above. For both data sets, a conditional inference tree and a random conditional inference forest are calculated.

#### 2.3.2. Model Validation

In order to validate the binary predictions (death or survival) obtained from the two different methods and sets of variables, we calculate the receiver operating characteristic (ROC) curve along with the associated AUC, the error rate, i.e., the percentage of wrong predictions, and the Brier score [27]. The Brier score is defined as the squared difference between the probability of the binary outcome and the actually observed one. This is a strictly proper scoring rule that is widely used to compare prediction models.

Calculating these measures for the predictions of the same data set that was used for developing the prediction models leads to, by far, too positive results, for which reason we use 10-fold cross-validation instead. This means that the data set is randomly distributed in 10 different random subsets. In a total of ten runs, one of these subsets (called folds) is taken out before the calculation of the trees and forests, respectively, leading to ten trees that can be different as the training set differs in each run. The one fold that was taken out before calculating its respective tree is then used for validating the predictions from the current run. The results of this cross-validation procedure are given as a summary of the ten different runs. We do not use leave-one-out cross-validation, because taking out just one measurement at a time leads to no relevant changes in the model that is derived from this data set. Note that for pragmatic reasons, all confidence intervals in the cross-validation are calculated using the usual formulae for proportions, means and AUC, although there is some debate in the literature if this really is appropriate due to dependencies in the data [28,29].

In addition to internal validation, where the original data set is used, we also perform external validation. This means that we apply the predictions from the model that was calculated based on the whole data set and validate its predictions in two completely new data sets. They stem from the Department of Anesthesiology, Intensive Care, Emergency and Pain Medicine, University Hospital Würzburg, Germany; and the Department of Anesthesiology, Intensive Care Medicine and Pain Therapy, University Hospital Frankfurt, Goethe University, Germany (Table 2). These data sets contain only the variables that were selected for the conditional inference trees. Based on the two trees, the predictions are calculated for both data sets separately and taken together. This allows for an assessment of the predictive abilities of the two trees in an external setting.

## 3. Results

A descriptive summary of the variables that were included in the small and the comprehensive data sets is given in Table 1 and Table S1.

**Figure 1:**
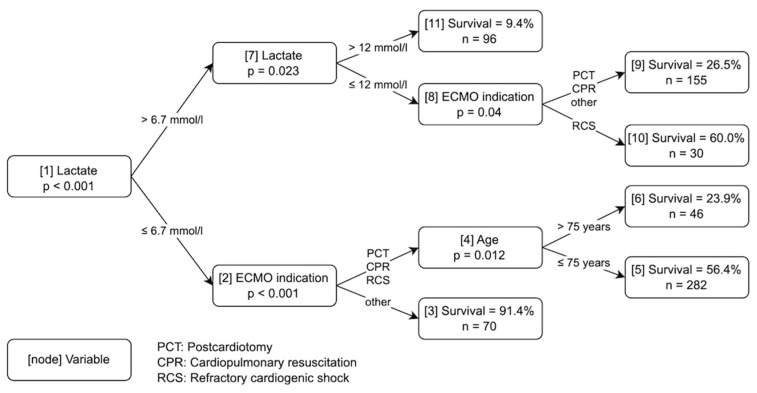
Decision Algorithm based on the Small Data Set. Numbers in square brackets represent the node numbers. Indications for ECMO therapy: postcardiotomy (PCT), cardiopulmonary resuscitation (CPR), refractory cardiogenic shock (RCS), and other. The category “other” included ECMO indications for lung transplantation and expansive thoracic surgery.

**Figure 2:**
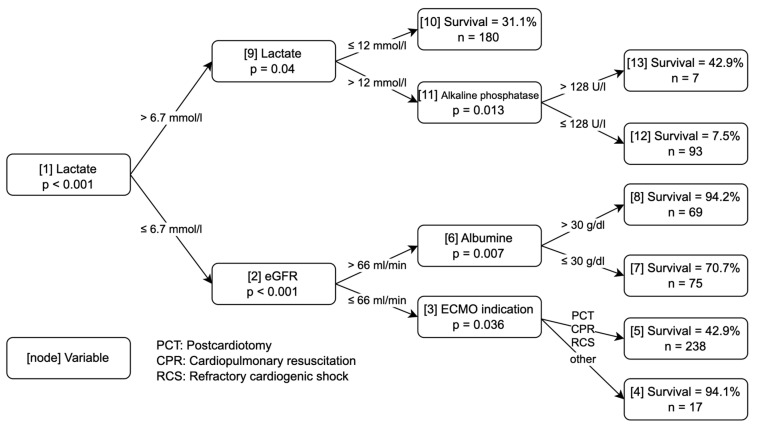
Decision Algorithm based on the Comprehensive Data Set. Numbers in square brackets represent the node numbers. Indications for ECMO therapy: postcardiotomy (PCT), cardiopulmonary resuscitation (CPR), refractory cardiogenic shock (RCS), and other. The category “other” included ECMO indications for lung transplantation and expansive thoracic surgery.

Figure 1 and Figure 2 show the two classification trees that were obtained for the two data sets. Each terminal node shows the survival probability for patients in this node, along with the total number of patients who ended up in this node. For the small data set, lactate, ECMO indication, and age were selected for the splits. In the comprehensive data set, lactate was selected for the first split, along with an additional lactate split as well as eGFR, ECMO indication, albumin, and alkaline phosphatase in the following nodes.

A closer look at the two different plots shows quite distinct predictions for the different paths of the plots: In the small data set, the outcomes of the outer nodes 3 and 11 are very clear, resulting in less than 10% deaths and more than 90% deaths, respectively. With respect to the remaining nodes, the predictions are not unambiguous, especially for nodes 5 and 10, with around 40% deaths in both cases.

A very similar situation can be seen in the tree from the comprehensive data set: several nodes show an outcome that is almost certain (nodes 4, 8, and 12), or at least more likely (nodes 7 and 10), whereas in two of the nodes no clear prediction is possible (nodes 5 and 13).

**Figure 3:**
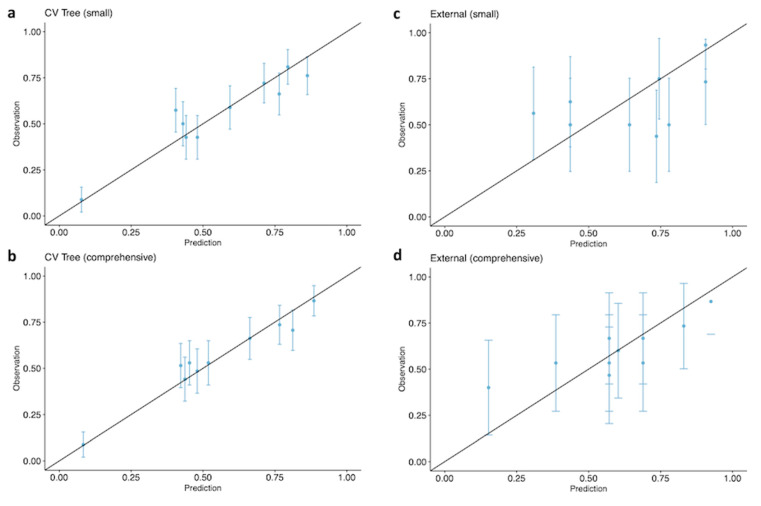
Calibration plots for ten-fold cross-validation. Left: derivation cohort (**a**) small and (**b**) comprehensive data set. Right: external validation variables (**c**) small and (**d**) comprehensive data set.

Ten-fold cross-validation of the binary predictions with conditional inference trees showed a cross-validated error rate of 35.8% (95% confidence interval from 32.3% to 39.5%) in the small data set. This means that in about 36% of the cases the prediction of death or survival was wrong, whereas the prediction matched the actually observed result in about 64% of the cases when the classification tree in Figure 1 was used. The associated area under the receiver operating characteristic (ROC) curve (AUROC) was 0.70 (95% confidence interval from 0.66 to 0.74). In the case of the comprehensive data set (Figure 2), the cross-validated error rate was practically the same, with a value of 35.4% (95% confidence interval from 31.8% to 39.0%), and the AUC had a value of 0.71 (95% confidence interval from 0.67 to 0.75). The cross-validated mean Brier scores of the two trees were 0.210 (95% confidence interval from 0.208 to 0.212) in the small data set and 0.211 (95% confidence interval from 0.209 to 0.212) for the comprehensive data set. Calibration plots for both data sets are shown in Figure 3. Satisfactory calibration can be seen for both trees.

Ten-fold cross-validation with forests showed a cross-validated error rate of 32.3% (95% confidence interval from 28.9% to 35.9%), an AUC of 0.75 (95% confidence interval from 0.72 to 0.79), and a mean Brier score of 0.199 (95% confidence interval from 0.198 to 0.201) in the small data set and of 32.0% (95% confidence interval from 28.6% to 35.6%), 0.76 (95% confidence interval from 0.72 to 0.79) and 0.198 (95% confidence interval from 0.197 to 0.200), respectively, for the comprehensive data set.

**Figure 4:**
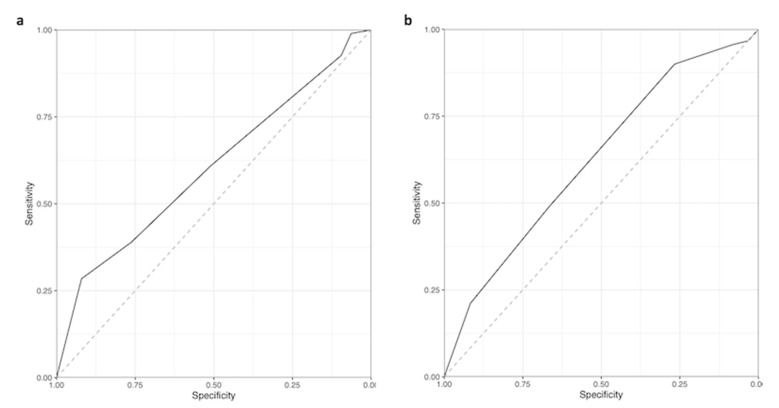
Receiver Operating Characteristic (ROC) curve (external validation) of the small ((**a**), left) and comprehensive ((**b**), right) decision trees. The predictions from the small tree (**a**) result in an Area Under the Curve (AUC) of 0.60 (95% confidence interval from 0.52 to 0.69), and the AUC from the comprehensive tree (**b**) is 0.63 (95% confidence interval from 0.54 to 0.72).

Table 2 shows the characteristics of the two validation data sets from Frankfurt and Wurzburg. The external validation resulted in an error rate of 43.0% (95% confidence interval from 35.6% to 50.8%) in the combined data set using the small tree and of 35.3% (95% confidence interval from 28.1% to 43.3%) using the comprehensive tree. This pattern is also reflected in the ROC curves (Figure 4): the predictions from the small tree result in an AUC of 0.60 (95% confidence interval from 0.52 to 0.69), and the AUC from the comprehensive tree is 0.63 (95% confidence interval from 0.54 to 0.72).

The results of external validation from the two different hospitals separately show differences between the two hospitals: predicting survival from the Wurzburg data only resulted in an error rate of 29.8% (95% confidence interval from 19.5% to 42.7%) and an AUC of 0.73 (95% confidence interval from 0.60 to 0.86) for the small tree, and an error rate of 24.1% (95% confidence interval from 14.6% to 36.9%) and an AUC of 0.74 (95% confidence interval from 0.60 to 0.89) for the comprehensive tree. 

The error rate for the small tree from the data from Frankfurt was 50.5% (95% confidence interval from 41.0% to 60.0%) and the AUC was 0.52 (95% confidence interval from 0.41 to 0.63), while the external predictions from the comprehensive tree resulted in an error rate of 41.7% (95% confidence interval from 32.3% to 51.7%) and an AUC of 0.56 (95% confidence interval from 0.45 to 0.67).

## 4. Discussion

This study demonstrates a machine learning predictive model for in-hospital mortality in patients receiving VA ECMO. In approximately 64% to 68% of cases, the prediction based on pre-ECMO variables matched the observed outcome, and the corresponding area under the receiver operating characteristics [ROC] curve [AUROC] values were 0.70 and 0.71. This compares favorably with the already established SAVE (Survival After Venoarterial ECMO) score, which has an AUROC of 0.68 [95% CI 0.64–0.71].

There were several different ECMO indications in our data set, which makes it relatively heterogeneous. However, this represents the typical clinical setting and makes the proposed algorithm more useable than if it was proposed for a very specific indication only. Despite the heterogeneity it performed well, which might be due in part to the higher sample size of the training set.

As the review by Eric. J. Topol [30] notes, the use of artificial intelligence is beginning to have an impact on predicting clinical outcomes that would be useful to healthcare systems. In the current literature, two studies report about applying machine learning to the ECMO cohort. Abbasi et. al. [14] compared classification and regression models to predict bleeding and thrombosis. The study cohort included 44 patients on ECMO. The most common indication was acute respiratory distress syndrome (59%), and 66% were supported with veno-venous ECMO. Rankings for variables varied and included ECMO indications, cannulation strategies, and duration. The study by Ayers et. al. [15] included 282 adult patients undergoing VA-ECMO. A deep neural network was trained to predict survival to discharge. The most important variables in predicting the primary outcome were lactate, age, total bilirubin, and creatinine. Their final model achieved high accuracy and a greater area under the curve than the SAVE score in predicting survival to discharge.

Typically, ECMO risk scores require many, and sometimes very detailed, variables. The calculation of the score is time consuming and the collection of the necessary variables requires the use of tools. In addition, the SAVE score [9] excludes ECMO during cardio-pulmonary resuscitation. The ENCOURAGE score [8] is specific for the population with acute myocardial infarction and cardiogenic shock, whereas the PREDICT score [11] refers to the prognosis after ECMO implantation.

We compared two different data sets: a small set with limited variables immediately available in the acute situation, and a large set with more comprehensive variables that are still easily available before the onset of ECMO. Prediction with conditional inference trees showed a similar error rate for the small and large data sets (35.79% vs. 35.35%). In the clinical setting, this finding is very helpful because the variables of the small set, namely age, lactate, and ECMO indication, are usually readily available. In an acute situation requiring the placement of an ECMO, clinicians are challenged to make a quick decision. Lactate can be determined at the point of care within minutes using blood gas analysis, and age and ECMO indication are obvious. In contrast, measuring blood samples in the laboratory requires much more time. Larger data sets have been shown not to improve the accuracy of the prediction [8]. Hence, awaiting the laboratory values such as albumin, alkaline phosphatase (ALP), and estimated glomerular filtration rate (eGFR) appears to be unnecessary. This shortens the time needed for decision making considerably.

Moreover, our suggested algorithm requires no computer and no training on how to use the respective programs, as it can be done using only a sheet of paper. This makes its application much more realistic and saves a lot of time compared to other predictions.

Consistent with the analyses by Abbasi et al. [14] and Ayers et. al. [15], we have shown that parameters such as age, ECMO indication, lactate, alkaline phosphatase, and creatinine or eGFR are suitable variables for the prediction of outcomes.

External validation showed that predictions for patients in the nodes with either a high probability of death or of survival can be very useful in clinical practice, whereas the predictions made for patients in the remaining nodes reflect the grade of uncertainty associated with the potential outcome. The scheme proposed in our analysis might serve as a new uncomplicated and rapid tool for the prediction of mortality in patients on ECMO immediately before implantation.

The differences between the two validation data sets are explained, at least in part, by the number of patients in each node. In Frankfurt, only about 14–15% are in the comparably certain nodes 3 and 11, whereas in Wurzburg, more than twice as many (39% and 33%) are located in this region (Supplementary Tables S2 and S3).

For both data sets, a conditional inference tree and a random forest were calculated. The error rates in conditional inference forests were slightly lower than in trees (32.3% vs. 35.79% for the small data set). However, the application of conditional inference forests is more complicated and time consuming, since predictions are made with a computer and cannot be obtained by hand using an easily understandable pencil-and-paper system. Therefore, the application of conditional inference forests is unsuitable in the acute situation.

### Limitations

Data were retrospectively collected which leads to potential bias. Over the long period of observation, there have been technological and procedural changes in ECMO therapy that may affect the chances of survival. Despite the established classification of ECMO indications in the literature, they may be interpreted and classified differently from center to center.

## 5. Conclusions

Conditional inference trees have the potential to contribute to prognostic clinical decision making for patients receiving ECMO therapy. They may provide a degree of accuracy in mortality prediction and prognostic stratification using readily available variables. Nonetheless, it is of utmost importance to further study the factors that influence the outcome in this complex situation.

## Figures and Tables

**Table 1:** Variables of the Small Data Set.

	Training
	N = 679
**Characteristics**	
Age, y	60 (49 to 69)
Sex, female	187 (28%)
BMI, kg/m^2^	25.7 (22.9 to 29.0) [8]
Indication	
Postcardiotomy	215 (31.7%)
Cardiopulmonary resuscitation	159 (23.4%)
Refractory cardiogenic shock	234 (34.5%)
Other	71 (10.5%)
In-hospital mortality	377 (56%)
Out-of-hospital resuscitation	40 (5.9%)
In-hospital resuscitation	119 (18%)
ROSC, min	35 (13 to 60)
**Comorbidities**	
Charlson comorbidity index	3.0 (2.0 to 5.0) [6]
Coronary artery disease	326 (48%)
Congestive heart failure	243 (36%) [1]
Peripheral vascular disease	118 (17%)
Cerebrovascular event	41 (6%)
Dementia	0 (0%)
Obstructive pulmonary disease	55 (8.1%)
Connective tissue disease	58 (8.5%)
Peptic ulcer disease	12 (1.8%)
Liver disease	12 (1.8%)
Diabetes mellitus	97 (14%)
Chronic kidney disease	53 (7.8%)
Solid tumor	50 (7.4%)
Leukemia	10 (1.5%)
Lymphoma	6 (0.9%)
AIDS	0 (0%)
**Blood Gas Analysis**	
Hematocrit, mmol/L	0.30 (0.25 to 0.38) [30]
Hemoglobin, g/L	97 (82 to 124) [28]
Lactate, mmol/L	5.6 (2.0 to 9.9) [83]
Base excess, mmol/L	−7.0 (−12.0 to −2.0) [85]
Bicarbonate, mmol/L	18.4 (14.5 to 22.0) [85]
Carbon dioxide partial pressure, kPa	5.11 (4.33 to 6.13) [84]
Oxygen partial pressure, kPa	16 (10.0 to 30.0) [13]
Glucose, mmol/L	8.8 (6.6 to 12.3) [82]
**Laboratory Values**	
eGFR, mL/min	53 (37 to 74) [10]
Albumin, g/dL	27 (20 to 33) [174]
Alkaline phosphatase, U/I	69 (49 to 100) [196]

Acquisition before VA-ECMO implantation. Data presents as median and IQR. Categorical variables present as number and percentage (%). If necessary, the number of missing data is indicated in parentheses [n]. Abbreviations: AIDS, Acquired Immune Deficiency Syndrome; BMI, body mass index; eGFR, estimated glomerular filtration rate; ROSC, Return of Spontaneous Circulation.

**Table 2:** Comparison of both validation data sets.

	Wurzburg	Frankfurt
	N = 57	N = 101
Age, y	57 (49 to 65)	70 (63 to 76)
Postcardiotomy	2 (3.5%)	60 (59.4%)
Cardiopulmonary resuscitation	31 (54.4%)	21 (20.8%)
Refractory cardiogenic shock	18 (31.6%)	16 (15.8%)
Other	6 (10.5%)	4 (4.0%)
In-hospital mortality	39 (68%)	56 (55%)
Lactate, mmol/L	9.3 (5.3 to 14.2)	6.0 (3.2 to 8.9)
eGFR, mL/min	50 (32 to 66) [2]	43 (32 to 60) [2]
Albumin, g/dL	25 (21 to 32) [1]	19 (15 to 24) [2]
Alkaline phosphatase, U/I	64 (45 to 118) [3]	42 (30 to 68) [5]

Data presents as median and IQR. Categorical variables present as number and percentage (%). If necessary, the number of missing data is indicated in parentheses [n]. Department of Anesthesiology, Intensive Care, Emergency and Pain Medicine, University Hospital Würzburg, Germany (Wurzburg); and Department of Anesthesiology, Intensive Care Medicine and Pain Therapy, University Hospital Frankfurt, Goethe University, Germany (Frankfurt). Abbreviation: eGFR, estimated glomerular filtration rate.

## Data Availability

The data are not publicly available due to privacy or ethical restrictions.

## Supplementary Materials

The following supporting information can be downloaded at: https://www.mdpi.com/article/10.3390/jcm12196243/s1, Table S1. Additional Training Variables of the Comprehensive Data Set; Table S2. Comparison of Validation Data Sets Small; Table S3. Comparison. Comparison of Validation Data Sets Comprehensive.

## References

[1] Eckman P.M., Katz J.N., El Banayosy A., Bohula E.A., Sun B., van Diepen S. (2019). Veno-Arterial Extracorporeal Membrane Oxygenation for Cardiogenic Shock: An Introduction for the Busy Clinician. Circulation.

[2] Pineton de Chambrun M., Bréchot N., Combes A. (2019). Venoarterial extracorporeal membrane oxygenation in cardiogenic shock: Indications, mode of operation, and current evidence. Curr. Opin. Crit. Care.

[3] Chung M., Zhao Y., Strom J.B., Shen C., Yeh R.W. (2019). Extracorporeal Membrane Oxygenation Use in Cardiogenic Shock: Impact of Age on In-Hospital Mortality, Length of Stay, and Costs. Crit. Care Med..

[4] Fernando S.M., Qureshi D., Tanuseputro P., Fan E., Munshi L., Rochwerg B., Talarico R., Scales D.C., Brodie D., Dhanani S. (2019). Mortality and costs following extracorporeal membrane oxygenation in critically ill adults: A population-based cohort study. Intensive Care Med..

[5] Chen W.C., Huang K.Y., Yao C.W., Wu C.F., Liang S.J., Li C.H., Tu C.Y., Chen H.J. (2016). The modified SAVE score: Predicting survival using urgent veno-arterial extracorporeal membrane oxygenation within 24 hours of arrival at the emergency department. Crit. Care.

[6] Kowalewski M., Zieliński K., Maria Raffa G., Meani P., Lo Coco V., Jiritano F., Fina D., Matteucci M., Chiarini G., Willers A. (2021). Mortality Predictors in Elderly Patients with Cardiogenic Shock on Venoarterial Extracorporeal Life Support. Analysis From the Extracorporeal Life Support Organization Registry. Crit. Care Med..

[7] Lee H.S., Kim H.S., Lee S.H., Lee S.A., Hwang J.J., Park J.B., Kim Y.H., Moon H.J., Lee W.S. (2019). Clinical implications of the initial SAPS II in veno-arterial extracorporeal oxygenation. J. Thorac. Dis..

[8] Muller G., Flecher E., Lebreton G., Luyt C.E., Trouillet J.L., Bréchot N., Schmidt M., Mastroianni C., Chastre J., Leprince P. (2016). The ENCOURAGE mortality risk score and analysis of long-term outcomes after VA-ECMO for acute myocardial infarction with cardiogenic shock. Intensive Care Med..

[9] Schmidt M., Burrell A., Roberts L., Bailey M., Sheldrake J., Rycus P.T., Hodgson C., Scheinkestel C., Cooper D.J., Thiagarajan R.R. (2015). Predicting survival after ECMO for refractory cardiogenic shock: The survival after veno-arterial-ECMO (SAVE)-score. Eur. Heart J..

[10] Wang L., Yang F., Wang X., Xie H., Fan E., Ogino M., Brodie D., Wang H., Hou X. (2019). Predicting mortality in patients undergoing VA-ECMO after coronary artery bypass grafting: The REMEMBER score. Crit. Care.

[11] Wengenmayer T., Duerschmied D., Graf E., Chiabudini M., Benk C., Mühlschlegel S., Philipp A., Lubnow M., Bode C., Staudacher D.L. (2019). Development and validation of a prognostic model for survival in patients treated with venoarterial extracorporeal membrane oxygenation: The PREDICT VA-ECMO score. Eur. Heart J. Acute Cardiovasc. Care.

[12] Schrutka L., Rohmann F., Binder C., Haberl T., Dreyfuss B., Heinz G., Lang I.M., Felli A., Steinlechner B., Niessner A. (2019). Discriminatory power of scoring systems for outcome prediction in patients with extracorporeal membrane oxygenation following cardiovascular surgery. Eur. J. Cardio-Thorac. Surg..

[13] Le Gall J.R., Lemeshow S., Saulnier F. (1993). A new Simplified Acute Physiology Score (SAPS II) based on a European/North American multicenter study. J. Am. Med. Assoc..

[14] Abbasi A., Karasu Y., Li C., Sodha N.R., Eickhoff C., Ventetuolo C.E. (2020). Machine learning to predict hemorrhage and thrombosis during extracorporeal membrane oxygenation. Crit. Care.

[15] Ayers B., Wood K., Gosev I., Prasad S. (2020). Predicting Survival After Extracorporeal Membrane Oxygenation by Using Machine Learning. Ann. Thorac. Surg..

[16] Sahli S.D., Kaserer A., Braun J., Halbe M., Dahlem Y., Spahn M.A., Rössler J., Krüger B., Maisano F., Spahn D.R. (2022). Predictors associated with mortality of extracorporeal life support therapy for acute heart failure: Single-center experience with 679 patients. J. Thorac. Dis..

[17] Alba A.C., Foroutan F., Buchan T.A., Alvarez J., Kinsella A., Clark K., Zhu A., Lau K., McGuinty C., Aleksova N. (2021). Mortality in patients with cardiogenic shock supported with VA ECMO: A systematic review and meta-analysis evaluating the impact of etiology on 29,289 patients. J. Heart Lung Transpl..

[18] Guglin M., Zucker M.J., Bazan V.M., Bozkurt B., El Banayosy A., Estep J.D., Gurley J., Nelson K., Malyala R., Panjrath G.S. (2019). Venoarterial ECMO for Adults: JACC Scientific Expert Panel. J. Am. Coll. Cardiol..

[19] Hothorn T., Hornik K., Zeileis A. (2006). Unbiased Recursive Partitioning: A Conditional Inference Framework. J. Comput. Graph. Stat..

[20] Hothorn T., Hornik K., van de Wiel M.A., Zeileis A. (2006). A Lego System for Conditional Inference. Am. Stat..

[21] Strobl C., Malley J., Tutz G. (2009). An introduction to recursive partitioning: Rationale, application, and characteristics of classification and regression trees, bagging, and random forests. Psychol. Methods.

[22] Buri M., Tanadini L.G., Hothorn T., Curt A. (2022). Unbiased recursive partitioning enables robust and reliable outcome prediction in acute spinal cord injury. J. Neurotrauma.

[23] Tanadini L.G., Steeves J.D., Hothorn T., Abel R., Maier D., Schubert M., Weidner N., Rupp R., Curt A. (2014). Identifying homogeneous subgroups in neurological disorders: Unbiased recursive partitioning in cervical complete spinal cord injury. Neurorehabilit. Neural Repair.

[24] Breiman L. (2001). Random forests. Mach. Learn..

[25] Hothorn T., Jung H.H. (2014). RandomForest4Life: A random forest for predicting ALS disease progression. Amyotroph. Lateral Scler. Front. Degener..

[26] Strobl C., Boulesteix A.-L., Zeileis A., Hothorn T. (2007). Bias in random forest variable importance measures: Illustrations, sources and a solution. BMC Bioinform..

[27] Rufibach K. (2010). Use of Brier score to assess binary predictions. J. Clin. Epidemiol..

[28] Bates S., Hastie T., Tibshirani R. (2023). Cross-validation: What does it estimate and how well does it do it?. J. Am. Stat. Assoc..

[29] Bradley A.A., Schwartz S.S., Hashino T. (2008). Sampling uncertainty and confidence intervals for the Brier score and Brier skill score. Weather. Forecast..

[30] Topol E.J. (2019). High-performance medicine: The convergence of human and artificial intelligence. Nat. Med..

