# Supplementary material for: Predicting Survival for Veno-Arterial ECMO Using Conditional Inference Trees—A Multicenter Study
Source: J Clin Med. 2023 Sep 28;12(19):6243. doi: 10.3390/jcm12196243 (PMC10573956; doi:10.3390/jcm12196243)
Supplement: Supplementary file 1 [file jcm-12-06243-s001.zip › jcm-2590714-supplementary.pdf]

Supplementary Material for:

Predicting Survival for Veno-Arterial ECMO Using Conditional Inference Trees—A Multicenter Study

<u>Content of additional tables:</u>

- **Supplementary Table S1. Additional Training Variables of the Comprehensive Data Set**

- **Supplementary Table S2. Comparison of Validation Data Sets Small**

- **Supplementary Table S3. Comparison of Validation Data Sets Comprehensive**

Supplementary Table S1. Additional Training Variables of the Comprehensive Data Set

	**Training Comprehensive** N = 679
**VA-ECMO Characteristics**	
External insertion [a]	62 (9.1%)
ECMO for Transplantation Tx	
insertion after Tx	57 (8.4%)
insertion during Tx	26 (3.8%)
insertion pre Tx	7 (1.0%)
**Comorbidities**	
SOFA score	11 (8 to 13) [361]
**Laboratory values**	
pH	7.30 (7.17 to 7.39) [84]
Platelets, G/l	172 (104 to 243) [8]
Leucocytes, G/l	11 (8 to 17) [9]
INR	1.4 (1.2 to 1.9) [11]
Partial thromboplastin time, sec	52 (32 to 134) [155]
thrombin time, sec	29 (18 to 120) [214]
Fibrinogen, g/l	2.6 (1.6 to 4.25) [32]
Coagulation factor V, %	36 (17 to 64) [216]
Coagulation factor XIII, %	65 (47 to 86) [145]
Urea, mmol/l	7.6 (5.8 to 11.0) [15]
Creatinine, µmol/l	116 (84 to 160) [10]
Bilirubin, µmol/l	13 (7 to 25) [146]
Lactate dehydrogenase, U/l	780 (473 to 1579) [47]
Aspartate aminotransferase, U/l	113 (40 to 428) [20]
Alanine transaminase, U/l	61 (24 to 265) [12]
Gamma-glutamyltransferase, U/l	39 (20 to 84) [104]
C-reactive protein, mg/l	17 (4 to 77) [16]
Creatine kinase, U/l	350 (127 to 862) [20]
Myoglobin, µg/l	542 (159 to 1466) [31]
Troponin T High Sensitive, ng/l	711 (149 to 2697) [84]
NTproBNP, ng/l	3592 (1107 to 11966) [292]

Beside all variables of Table 1 these additional variables were used in the comprehensive training data set. Data presents as median and IQR. Categorical variables as number and percentage (%). If necessary, the number of missing data indicates in parentheses [n].

[a] ECMO insertion by University Hospital Zurich outreach team in another hospital before transfer to University Hospital Zurich for definitive care.

Abbreviations: INR, International Normalized Ratio; NTproBNP, N-terminal pro-B-type natriuretic peptide; SOFA, Sepsis-related organ failure assessment score

Supplementary Table S2. Comparison of Validation Data Sets Small

	**Node**	3	5	6	9	10	11	Total
**Center**								
Würzburg		2 (3.5%)	20 (35.1%)	1 (1.8%)	8 (14.0%)	5 (8.8%)	21 (36.8%)	57 (100%)
Frankfurt		3 (3.0%)	36 (35.6%)	19 (18.8%)	29 (28.7%)	3 (3.0%)	11 (10.9%)	101 (100%)
Total		5 (3.2%)	56 (35.4%)	20 (12.7%)	37 (23.4%)	8 (5.1%)	32 (20.3%)	158 (100%)

Supplementary Table S3. Comparison of Validation Data Sets Comprehensive

	**Node**	4	5	7	8	10	12	13	Total
**Center**									
Würzburg		1 (1.9%)	12 (22.2%)	6 (11.1%)	4 (7.4%)	12 (22.2%)	13 (24.1%)	6 (11.1%)	54 (100%)
Frankfurt		3 (3.1%)	43 (44.8%)	10 (10.4%)	1 (1.0%)	28 (29.2%)	11 (11.5%)	0 (0.0%)	96 (100%)
Total		4 (2.7%)	55 (36.7%)	16 (10.7%)	5 (3.3%)	40 (26.7%)	24 (16.0%)	6 (4.0%)	150 (100%)
